# Behind the Counter

**Published:** 1893-02

**Authors:** 


### BEHIND THE COUNTER.

No. 2.

The morning of the second day I was at my post bright and early, and after occupying my peg, I proceeded to put things in order in my department, when my superior put in an appearance and complimented me upon my aptitude in the business. Her few words of commendation gave me great encouragement, and I could but reflect that much of our successes in life are due to some "little word in kindness spoken" at the moment of greatest need.

My superior then called me to her side and placing in my hands a regulation sales book, remarked, "This is yours, and this is you," pointing to the figures 91 below the word "basement," on the cover. "We all hang our baptismal names on our peg with our hats and wraps," said she, "to resume them on leaving. You will call me 13, the unlucky number not altogether misplaced."

In my intervals of leisure I busied myself with making a nearer acquaintance with the multitudinous wares committed to our charge, and felt no little pride on making my first sale, and committing it with my leaflet memorandum to the active little messenger girl who responded to my authoritative call of "Cash! cash!"

Soon afterwards there was a demand for 91 to repair to the accounting desk. On presenting myself I was shown my long column of figures, and told to add them up and set the total plainly underneath. This in my haste I had neglected to do, being over anxious to complete the transaction, unassisted by No. 13, who was temporarily absent, but being a raw hand I was not subjected to a fine to which I had laid myself liable under the rules. Indeed, I soon learned there was a system of fines for minor offences and misfeasances, covering so many points that their unsparing application sometimes consumed the greater share of the unfortunate's weekly salary. Gathering in knots and whispering or giggling was an infraction of rules most commonly infringed by the younger members. Quoting from my note book of this date, I read as follows: "We had quite a commotion at our bargain counter to-day. A woman made a small purchase, and on looking for her money, declared she laid it on the table for me to send with her purchase to the package room, and on being assured that I had not seen it, accused a lady who stood near her of its theft. She made a great time over her supposed loss, which brought that much dreaded individual, the floor walker, into requisition, and whilst he was hearing her story there was a metallic ring upon the floor. It proved to be her stray half-dollar, which had somehow secreted itself in her clothing." It is needless to say that both the accused lady and I were fully exonerated, but my customer was so overwhelmed by her own witness, that with a significant toss of her head, she left the seat of war without completing her nine cent purchase. I afterwards learned that such scenes were not unfrequent.

Before leaving the store on my second day, I was furnished with a tell-tale key as we call it, which on being inserted into its appointed clock-work monitor, registers the exact time of its possessor's morning appearance, a duty enjoined upon all peg holders. There is no lack of daily prayers for this much abused machine to retire from business. I was informed that there were four of them, accounting from the numeral one to some fifteen hundred. Some of the more profane among the male help have dubbed it "the little cuss," and now and then one is discharged for tampering with it. PAULINE.

